# Parasitological and molecular diagnosis of cutaneous leishmaniasis among indigenous peoples in the state of Roraima, Brazil.

**DOI:** 10.1590/0037-8682-0006-2020

**Published:** 2020-10-21

**Authors:** Joseneide Viana de Almeida, Cristian Ferreira de Souza, Isabela de Oliveira Teixeira, Hugo Oswaldo Valdivia, Daniella Castanheira Bartholomeu, Reginaldo Peçanha Brazil

**Affiliations:** 1 Universidade Estadual de Roraima, Boa Vista, RR, Brasil.; 2 Instituto Oswaldo Cruz, Laboratório de Doenças Parasitárias, Rio de Janeiro, RJ, Brasil.; 3 Universidade Estácio de Sá, Rio de Janeiro, RJ, Brasil.; 4 Universidade Federal de Minas Gerais, Instituto de Ciências Biológica, Departamento de Parasitologia, Belo Horizonte, MG, Brasil.

**Keywords:** Cutaneous leishmaniasis, Diagnosis, Indians

## Abstract

**INTRODUCTION:**

We diagnose cases of cutaneous leishmaniasis (CL) among indigenous peoples of the state of Roraima, Brazil, and discuss some aspects of its epidemiology.

**METHODS:**

Skin imprints, and lesion exudate samples collected on filter paper were examined using parasitological and molecular techniques, respectively.

**RESULTS:**

Of 30 indigenous individuals, representing several ethnic groups, with suspected cases of CL, 27 (90%) tested positive for *Leishmania* spp. by PCR, and 21 (70%) by parasitological microscopy.

**CONCLUSIONS:**

Cutaneous leishmaniasis is indistinctly present among indigenous peoples from different regions of the state of Roraima. Individuals from seven of the ten existing ethnic groups in the state tested positive for CL, demonstrating the need for further investigation of the disease among these ethnic groups.

## INTRODUCTION

Leishmaniases are neglected infectious diseases transmitted by the sand fly (Diptera: Psychodidae: Phlebotominae)^1^ ^,^ ^2^ that occur in the poorest countries among the most vulnerable populations with limited access to health services. Leishmaniases display worldwide distribution, with most cases occurring in Africa, Asia, and the Americas. In the Americas, leishmaniasis is present in 18 countries, and the most common clinical form is cutaneous leishmaniasis (CL). In addition, mucocutaneous leishmaniasis (MCL) displays chronic progression that may lead to deformities and long-term effects^3^, while visceral leishmaniasis (VL) is more severe, and often fatal if left untreated. Most cases occur in Brazil, East Africa, and India. An estimated 50,000 to 90,000 new cases of VL occur worldwide annually, with only 25 to 45% reported to the WHO. Visceral leishmaniasis remains one of the most prevalent parasitic diseases, with outbreaks and potential mortality^4^. In South America, Brazil is one of most endemic regions for VL and CL^5^. 

In the Brazilian Health System (SUS - Sistema Único de Saude), leishmaniasis represents a complex of diseases with clinical aspects and epidemiological diversity that need to be better studied, and that are considered to be a major public health problem^6^. The incidence of CL has been increasing in recent years, with an average of 35,000 cases/year distributed from the southern Amazon Basin to the southernmost point of the country^7^ ^,^ ^8^ ^,^ ^9^ ^,^ ^4^ taking the form of epidemic outbreaks due to forest clearing, logging, and human activities linked to agriculture and leisure^10^.

In the Americas, visceral leishmaniasis (LV) is caused by *Leishmania infantum*, but the cutaneous form can be caused by at least 12 species of *Leishmania* that infect humans and animals. In Brazil, seven species of the genus *Leishmania* have been identified. Six of these belong to the subgenus *Viannia,* and one to the subgenus *Leishmania* ^11^. 

The State of Roraima is included within an area of the Amazon rainforest that overlaps nine Brazilian states. These states belong to the northern region, with municipalities that cover large, difficult to access territories, and are unable to adopt measures recommendeted by the MoH to control the spread of CL. Indigenous communities appear to be especially vulnerable, because they occupy highly endemic areas for CL with limited access to health services^12^.

The state of Roraima exists within this region and fits the scenario described above that we planned to study. has been suffering profound environmental changes in recent years, including occupation of forest áreas, and plowing for mining, agriculture, and raising livestock. These environmental disturbances may be contributing to maintenance of the leishmaniasis cycle in this state. A recent study describes the epidemiological profile of CL in the state of Roraima between 2007 and 2016^13^, which shows a 13% incidence of CL among indigenous populations, demonstrating a need for further studies to understand the endemicity among these specific populations in the state.

## METHODS

### Study area and populations

**FIGURE 1:**
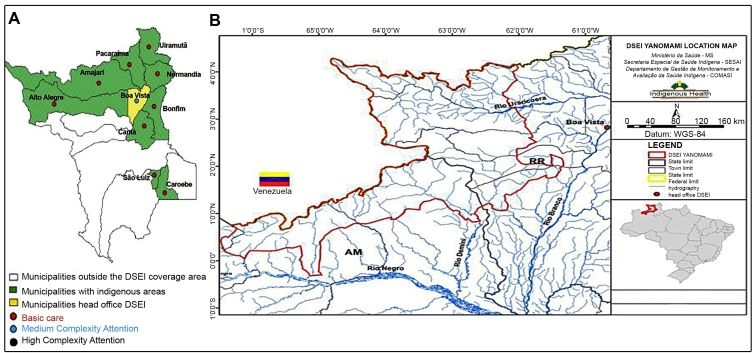
DSEIS of Roraima. **A:** Map of the state of Roraima with municipalities covered by DSEI East, and **B:** Map location of DSEI Yanomami. **Source: Fig. A:** FUNASA^17^, 2010; **Fig. B:** BRAZIL, 2017^18^.

Roraima obtained statehood in 1988, and covers an area of 225,116.1 km, bordered on the north by Guyana and the Republic of Venezuela, on the south by the states of Amazonas and Pará, on the east by the Cooperative Republic of Guyana; and on the west by the State of Amazonas and the Republic of Venezuela. The total length of international border spans 1922 km^14^. In 2019, Roraima had an estimated population of 605,761 inhabitants, factoring in estimated Venezuelan migration^15^. According to data from the Roraima Indigenous Council (CIR)^16^ in 2017, the state's indigenous population was 53,990 peopleYanomami (which has the largest estimated population, at 25,700 people), Ingarikó, Taurepang, Macuxí, Waimiri- Atroari, Wapixana, Wawai, Yekuana, Patamona, and Sapará. Also according to the CIR, the indigenous population comprises 33 communities: Ananás, Anaro, Aningal, Anta, Araçá, Arapuá, Barata, Livramento, Bom Jesus, Boqueirão, Cajueiro, Canauanim, Jabuti, Jacamim, Malacacheta, Mangueira, Manoa/Pium, Moskow, Muriru, Ouro, Pium, Ponta da Serra, Raimundão, Raposa Serra do Sol, Santa Inez, São Marcos, Serra da Moça, Sucuba, Tabalascada, Trombetas/Mapuera, Truaru, Waimiri-Atroari, Wai-wai and Yanomami. The health care of these peoples is the designated responsibility of SESAI - RR, which is represented by two DSEIS systems in Roraima: DSEI Yanomami, and DSEI East^17^ ^,^ ^18^ (Figure 1).

### Collection of data from SINAN and samples

An epidemiological survey of cutaneous leishmaniasis was conducted among the indigenous populations of Roraima, through retrospective analysis of cases reported in Sinan from 2013 to 2017^19^. From 2017 to 2018, samples were collected (by lesion scarification) for parasitological examination. Concomitantly, samples of scraped material were collected on filter paper (FTA® cards) for further characterization of *Leishmania* species by molecular tools.

### Parasitological Examination

Direct examination was performed by specimen collection from the edge of the ulcerated lesion (scarification) using aseptic technique with a lancet and/or a sterile scalpel. The collected material was smeared on slides, fixed with metanol, and stained with Giemsa and/or Panotic. Slides were observed by optical microscopy at 100X magnification.

### Molecular Detection

Samples of cells, tissues, and blood collected from lesion scarification of each patient with suspected LT were identified and subjected to DNA extraction using a Gentra Puregene® Cell and Tissue Extraction Kit (QIAGEN, Hilden, Germany) , following the manufacturer's protocol. 

The presence of *Leishmania* DNA was detected by PCR using subgenus-specific primers that target *Leishmania* or *Viannia* minicircle-kinetoplast (kDNA) DNA (Table 1). Species within each subgenus will be defined in due course by sequencing positive material obtained by PCR.

**TABLE 1:** Primers used in PCR reactions with their respective sequences and amplicon sizes.

Target	Primer	Sequence	Amplicon Size
**Subgenus Leishmania**	kDNA.Leish.F	5’CGTGGGGGAGGGGCGTTCT 3’	135 bp
	kDNA.Leish.R	5’CCGAAGCAGCCGCCCCTATT 3’	
**Subgenus Viannia**	MP1L	5’ TACTCCCCGACATGCCTCTG 3’	70 bp
	MP3H	5’ GAACGGGGTTTCTGTATGC 3’	

Source: Adapted from Cardoso *et al* 2019^5^.

PCR reactions were performed in a total volume of 20 μL, containing 1X GoTaq green buffer (Promega), 0.2 mM dNTPs, 0.5 μM of each primer, 1 U of DNA Taq polymerase (Phoneutria) and 1 or 5 μL of DNA. PCR reactions templated with samples on filter paper were performed in two stages. Initially, samples were analyzed in 5 patient pools with primers specific to the subgenera *Leishmania* and *Viannia*. In this initial screen, 5 μL of each pool was used per PCR reaction. Subsequently, individual DNA samples from patients in each positive pool were subjected to PCR using subgenus *Leishmania* and *Viannia* primers. Thermal cycle conditions consisted of an initial denaturation at 94°C for 5 min; followed by 30 cycles of denaturation at 94°C for 30 s, annealing at 56°C for subgenus *Leishmania*, and 60°C for subgenus *Viannia* for 30 s, and extension at 72°C for 30 s; followed by a final extension step at 72°C for 7 min. Expected band size for each reaction is indicated in Table 1. The amplified products were analyzed by electrophoresis in 2.0% agarose gels in 1x TAE buffer containing 0.5 μg/mL ethidium bromide, and visualized under UV light using ImageQuant LAS 4000 (GE Health Life Sciences).

### Ethical considerations

This study was approved by the Research Ethics Committee of the Federal University of Roraima under CAAE 57445116.3.0000.5302.

## RESULTS

During the study period, 313 confirmed cases of CL among indigenous people in the state of Roraima were reported by SINAN^19^. The year 2016^20^ had the lowest number of cases, although levels were similar in other years, except for 2015, in which 94 cases were reported (Table 2). From 2016 to 2018, 30 samples were collected from the study population for parasitological examination and molecular diagnosis. Twenty one (70%) were positive, and 9 (30%) were negative. Twenty seven (90%) of these PCR-kDNA samples for *Leishmania* species of the subgenus *Viannia* were positive (Table 3). All samples were negative for the subgenus *Leishmania*. All patients developed the cutaneous form, and presented with the following distribution of numbers of lesions: 22 (73%) patients had a single lesion, 8 (27%) had two or more lesions distributed throughout the body. Twenty two (73%) of the CL patients were male, and 8 (27%) were female (Table 3).

**TABLE 2:** Distribution of LC cases among indigenous peoples in Roraima, Brazil, from 2013 to 2017.

Year	Number of Cases
2013	60
2014	59
2015	94
2016	49
2017	51
**Total**	**313**

Source: SINAN 2019.

**TABLE 3:** Demographics of LC cases among indigenous peoples, and diagnostic results of samples collected on PCR filter paper and slide imprints for microscopic examination in Roraima, Brazil, from 2016 to 2018.

Patients			Number of Lesions		Results	
Variables		Sampling	One	Two or More	Positive by Microscopy	Positive for KDNA **
Gender	Male	22	15	7	15	20
	Female	8	7	1	6	7
Age	0 a 10	2	2	0	2	2
	11 à 20	10	7	3	5	9
	21 à 30	8	5	2	7	9
	31 à 40	5	3	3	5	3
	41 à 50	3	3	0	1	2
	51 e +	2	2	0	1	2
Ethnicities	Ianomami	11	8	3	11	11
	Sanumã	4	3	1	2	4
	Macuxi	5	3	2	4	5
	Yekuana	4	3	1	1	3
	Xiriana	3	2	1	1	2
	Waiwai	1	1	0	1	1
	Igaricó	2	2	0	1	1

**. Positive samples for presence of KDNA with *L. Viannia* MP1L - F Primer and MP3H - R kDNA. Amplicon: 70bp.

Table 3 displays an evaluation of patient data by age group and year. The highest percentage of cases occurred in patients aged 21-30 years (26.67%) followed by the 31-40 year old cohort (16.67%).

The ethnic distribution of kDNA examination subjects was as follows: 11 (40.74%) patients were Ianomami, 4 (14.81%) Sanumã, 5 (18.52%) Macuxi, 3 (11.11%) Yekuana, 2 ( 7.4%) Xiriana, 1 (3.7%) Wai-wai and 1 (3.7%) Igaricó. Ianomami and Macuxí people are more numerous in the state according to the Roraima Indian Council^16^, and thus have more individuals at risk of contracting CL.

Regarding the distribution of cases by municipality (Figure 1), we noticed that almost all cases concentrate in the municipalities of Uiramutã, Alto Alegre and Amajari, validating these results because these municipalities harbor the largest indigenous populations in the state.

## DISCUSSION

The results of this research are similar to data from a study on the distribution of cutaneous leishmaniasis cases in the municipality of Rio Preto da Eva, in the state of Amazonas^21^. However, these results are superior to the results obtained in characterization of *Leishmania* species in biological CL samples from patients in Brasiléia in the state of Acre^22^, where samples analyzed by PCR-kDNA were able to detect *Leishmania* DNA in 66.6% of patients.

Our results are consistent with case data from the general population published by the State Secretariat of Health of Roraima^23^ and the latest figures presented in reports

on the distribution of CL in the Americas up to 2019^1^. The high incidence of CL in males has been attributed to their increased contact with forest regions, during excursions into the forest to work and/or for leisure activities. The low incidence in females may be related to peridomiciliary and intradomiciliary transmission^6^. In the specific case of indigenous people, who, despite growing numbers of individuals changing their normal activities, maintain their lifestyle of exploring the forest in a “coivara” system , hunting, fishing, and gathering. This way of life results in a unique daily dynamic, characterized by daily forays into forest areas, capoeiras, fields, igapós, and streams^24^, which may favor transmission of the parasites responsible for CL.

The large number of CL cases in the age groups spanning 20-40 years is due to this group providing the largest proportion of the active work force in the field for hunting and fishing. Theoretically, these people are most exposed to the sand fly vector, while the lowest infection rates are seen in people above this age range that do not frequently participate in such activities. It is also noteworthy that CL cases occur among the age group between 7 months and 10 years, which is suggestive of intradomiciliary or peridomiciliary transmission among indigenous people who live in their own homes on small properties, such as the Macuxi, who live in small existing houses within the indigenous área, and attend schools located in their own territory. Transmission of CL among indigenous peoples living in the forested areas of Roraima suggests that these young people and children are being taken by their guardians to work áreas, or are participating in hunting and fishing, where they are exposed to phlebotomine, and can then be infected by CL causing parasites, as has been reported in a study of a region of Brasiléia in Acre^22^, where phlebotomine exposure was associated with CL transmission among people living in rural areas and working in rubber extraction.

It is noteworthy that a number of cases were found in Boa Vista, despite a report from the State Department of Health (2018)^23^ indicating that Boa Vista has no record of autonomous cases of CL. This discrepancy may suggest that some indigenous people are moving to the state capital for CL diagnosis and treatment. We also note that the municipality of Caroebe, located in the extreme south of the state on the border with Pará State, and the municipality of Pacaraima located in the extreme north of the state, bordering Venezuela, also reported cases of CL, demonstrating that the endemic disease is indistinctly presente among indigenous peoples in all municipalities within the state where these peoples live.

The State of Roraima, specifically among indigenous communities, has factors favorable to disease endemicity, necessitating further studies, focused on the transmission cycle, vectors, hosts, measures for prevention and control of LC in young people, and actions based on effective public policies for control of LC in Roraima.
